# Effects of Stumping on Ecological Stoichiometry and Allometric Growth in Leaf, Absorptive Root, and Rhizosphere Soil of *Hippophae rhamnoides*

**DOI:** 10.3390/plants14101513

**Published:** 2025-05-19

**Authors:** Lu Liu, Yuefeng Guo, Wangsuo Liu, Darifu Ba, Fei Feng

**Affiliations:** 1 Department of Chemistry and Environmental Engineering, Hetao University, Bayannur 015000, China; liuwangsuo@sina.com (W.L.); badarifu11@126.com (D.B.); 2 College of Desert Control Science and Engineering, Inner Mongolia Agricultural University, Hohhot 010018, China; 3 Bayannur City Water Conservancy Affairs Service Center, Bayannur 015002, China; fengfei0526@126.com

**Keywords:** stumping, stoichiometric characteristics, allometric relationship, allocation strategy, *Hippophae rhamnoides*

## Abstract

To clarify the effects of stumping on the C, N, and P allocation strategy of *Hippophae rhamnoides* L. artificial forests at the decaying stage in feldspathic sandstone areas, we tested stumping heights of 0, 10, 15, and 20 cm from the ground (denoted H1, H2, H3, and H4, respectively) with non-stumped trees as a control (CK). The N (LN, RN), P (LP, RP), and N:P (LN:LP, RN:RP) in the leaves and absorptive roots and the C, N, C:N, C:P, and N:P in rhizosphere soils after different treatments all manifested in the order H3 > H2 > H1 > H4 > CK. Among them, the LN and RN of H3 presented the largest amplitudes of increase (31% and 263%, respectively) compared with those of CK. There were very significant allometric relationships between LC and RC (−0.57, trade-off relationship), between LN and RN, and between LP and RP (0.32, 2.01; synergistic relationship) in stumped *H. rhamnoides*, and the accumulation rates of LC and LN were slower than those of RC and RN. After the stumping, certain correlations were present between the characteristics, except that neither LC nor RC significantly differed across the different treatments. The growth of *H. rhamnoides* after the different treatments was mainly regulated by P. The stumped *H. rhamnoides* grew at a faster rate, and the optimal stumping height was 15 cm. These findings are valuable for revegetation and for the prevention and control of soil erosion in feldspathic sandstone areas.

## 1. Introduction

Ecological stoichiometry refers to the balance of multiple chemical elements during ecological interactions, and it reflects the element contents and energy flow characteristics in the ecosystem [1,2]. Carbon (C), nitrogen (N), and phosphorus (P) are key nutrient elements in plant growth and development, and the ratios between them reflect a plant’s growth rate, nutrient utilization strategies, and restrictive elements [3,4]. Leaves are the main organ for the production and storage of substances in plants. The stoichiometry of elements in a plant’s leaves reflects its growth state and nutrient utilization efficiency; for example, leaf C reveals a plant’s photosynthetic ability and organic matter accumulation level [5]. Fine roots are the main organ of nutrient transportation [6], and absorptive roots are the fine roots that absorb water and nutrients [7]. The responses from the traits of absorptive roots to changes in the external environment more sensitively reflect variations in plants’ underground resource acquisition strategies. The rhizosphere is where roots most frequently come into contact with soils and microbes, and the rhizosphere environment directly affects the transfer and absorption of soil nutrients by the roots [8]. In particular, the availability of N and P in rhizosphere soil determines a plant’s productivity [9]. Thus, studying the allometric growth relationship between leaves and absorptive roots, along with the stoichiometric characteristics of C, N, and P in plants and rhizosphere soils, is significant for understanding plants’ nutrient characteristics and resource utilization strategies.

The allometric growth relationship is a basic theory for describing resource allocation. The physiological functions, nutrient absorption, and accumulation abilities of plants all differ among different organs. A plant’s response to its environment results from the joint response of all its organs. Such a response coordinates nutrient contents at a relatively stable nutrient ratio among the organs and maintains the normal growth needs of the body [10,11,12]. The allometric relationship can quantify a trade-off, reflecting adaptive strategies and optimizing the allocation of finite resources, or synergy, reflecting mutual cooperation between functions or traits, occurring in plants during environmental adaption [13,14]. There has been little research on the allocation of nutrient resources to the leaves and absorptive roots. Hence, studying the allometric growth relationship between leaves and absorptive roots can provide insights into plants’ growth strategies and adaptation mechanisms.

The feldspathic sandstone zone of Inner Mongolia is among the regions suffering the most severe soil erosion on the Loess Plateau and even worldwide. Feldspathic sandstones are under low diagenesis but become mud upon watering and turn into sand under strong winds. This zone has sparse natural vegetation and is suffering severe erosion, which complicates its administration. *Hippophae rhamnoides* L. is an important soil and water conservation plant in arid and semiarid areas. With its drought resistance, well-developed roots, and strong tillering and germination ability, it can propagate quickly. It has excellent soil- and water-preserving abilities and is very dominant in the feldspathic sandstone zone of Inner Mongolia. However, the growth and productivity of artificial forests of *H. rhamnoides* in this region decrease massively when they reach 10 years old [15,16], indicating that effective conservation is needed at this age. Reportedly, stumping can alter the functional traits of plants so that they can re-allocate resources and compensatorily recover growth, preventing growth decay. However, the sprouting effect of stumping is affected by multiple factors, of which the stump height is controllable [17].

At present, the research on stumping is mostly limited to analyses of the responses of cutting tree stools and sprouting branches, especially the responses of the above-ground biomass and functional traits to stumping [18,19,20]. However, there have been few studies on the properties of fine roots in plants, and no studies have been reported on the impact on absorptive roots and their nutrient element distribution. The allocation strategies of nutrient elements in the leaves, absorptive roots, and rhizosphere soils, as well as their interactions, in stumped *H. rhamnoides* in the feldspathic sandstone zone of Inner Mongolia are still unclear.

In view of the above, this study aims to analyze the influence of stumping heights on the stoichiometric ratios in the leaves, absorptive roots, and rhizosphere soil of *H. rhamnoides* and to explore the distribution and allometric relationship of nutrient elements in the leaves and absorptive roots of *H. rhamnoides* after different treatments. The correlations among nutrients in the leaves, absorptive roots, and rhizosphere soils, as well as the adaptation strategy to stumping, are further revealed. Through this study, we expect to understand the nutrient allocation strategies of *H. rhamnoides* before and after stumping and to find the stumping height that is suitable for vegetation recovery. The findings offer a theoretical basis and technical support for the management and sustainable vegetation operation of *H. rhamnoides* forests.

## 2. Materials and Methods

### 2.1. Study Area

**Figure 1:**
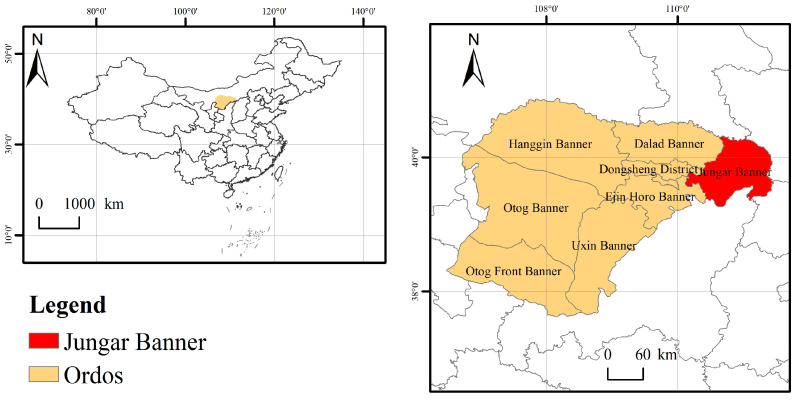
Geographical position of the study area.

The study area is located in the feldspathic sandstone-zone soil–water conservation science demonstration plot in Nuanshui Village, Jungar Banner, Ordos, Inner Mongolia (Figure 1). This area (39°42′–39°50′ N, 110°25′–110°48′ E) enjoys a temperate continental climate: long, dry winters and short, warm summers, and an average yearly temperature of 7.2 °C. Rainfall is mainly concentrated in the summer (June–August), with an average annual summer rainfall of 256.4 mm, accounting for 64.1% of the total average annual rainfall of 400 mm. This area enjoys sufficient sunshine, with an illumination duration of between 3100 and 3200 h, an illumination rate of over 70%, and an average annual frost-free period of 135 days every year. The regional soil is chestnut calcium soil, and the upper layer of the soil also contains small amounts of loessial soil and sand soil. Natural vegetation is sparse, and artificial vegetation is dominant in the region. The vegetation types are mostly shrubs and perennial herbs, including *H. rhamnoides*, *Pinus tabuliormis*, *Caragana korshinskii*, *Medicago sativa*, *Heteropappus altaicius*, and *Armeniaca sibirica*.

### 2.2. Experimental Design

In the demonstration plot, wintering artificial *H. rhamnoides* forests with basically consistent site conditions and forest compositions were chosen as experimental sites. The forests were positioned on a northwest-facing slope at a 4° angle. Trees were planted on the same slope face with row spacing of 2 m × 4 m, and they were stumped in early March 2020. The stumping heights were 0, 10, 15, and 20 cm above the ground (named treatments H1, H2, H3, and H4, respectively). An artificial forest of *H. rhamnoides* without stumping was set as the control (CK). All sites were 50 m × 50 m in area, and each treatment was tested in triplicate. Stumping was conducted using electric saws and pruning shears, which ensured that the incisions were flat and smooth, without burrs. The complete stumping mode was adopted. To decrease moisture loss, we painted the trees after stumping. In mid-August 2024, 5 clusters of healthy *H. rhamnoides* under basically consistent conditions from each sampling site were randomly chosen for measurements of their leaves, roots, and soils. In total, 75 clusters were selected.

### 2.3. Sample Collection and Processing

Healthy and mature leaves were collected in 4 directions (east, west, south, and north) from the wood canopy of each standard tree, and 10 medium-sized healthy leaves were harvested from each cluster of *H. rhamnoides*. At the same time, the root tracking method was used to collect absorptive roots under the same standard wood. Two points were randomly selected at the base of the standard wood; then, surface weeds, dead leaves, and other debris were removed, and the soil around the rhizosphere was carefully removed using a shovel. Soils were dug along the growing direction of lateral roots until root branches were reached. Digging continued along the branches until the root ends were reached. During the sampling, the loss of terminal low-grade roots was avoided as much as possible to ensure root completeness. After that, the terminal low-quality roots, i.e., the absorptive roots, were selected [21]. Rhizosphere soils were collected using a shaking method [22,23]. From the root soils left after the collection of the absorptive roots, large pebbles and animal and plant residues near the roots were discarded, and surface grains and impurities were shaken off the roots. Then, the rhizosphere soils attached within 4 mm of the root surface were collected. The samples of leaves, absorptive roots, and rhizosphere soils collected from the same tree were mixed separately, then put into labeled self-sealed bags, stored at a low temperature, and brought back to the laboratory.

The collected leaves and absorptive roots were washed with deionized water. Each sample of leaves, absorptive roots, or rhizosphere soils was divided into 2 parts for the detection of C/N and P contents. First, all the samples of leaves and absorptive roots were placed in an oven at 60 °C and dried to a constant weight. Then, a portion of each rhizosphere soil sample (1/2 of each sample) was put in the oven at 105 °C and dried until it reached a constant weight. The dried samples of leaves, absorptive roots, and rhizosphere soils were crushed using a PULVERISETTE 5 high-throughput ball milling system (Fritsch, Munich, Germany). After the samples were passed through a 0.149 mm sieve, 1/2 of each leaf or absorptive root sample was placed in an elemental analyzer to detect its C and N contents. The remaining 1/2 of each leaf or absorptive root sample was used to measure the P content using the H_2_SO_4_–H_2_O_2_ boiled Mo/Sb colorimetric method. The other 1/2 of each rhizosphere soil sample was dried naturally for one month. Animal and plant residues, lime grains, and stones were removed, and the remaining sample was ground with a stone grinding rod and passed through a 0.149 mm sieve. Then, the P contents in the rhizosphere soils were measured using the H_2_SO_4_–H_2_O_2_ boiled Mo Sb colorimetric method. Finally, the C, N, and P ecostoichiometric ratios in the leaves, absorptive roots, and rhizosphere soils were calculated separately.

### 2.4. Data Analysis

Data were analyzed using SPSS 26.0. The measured C, N, and P contents and C:N, C:P, and N:P stoichiometric ratios were used to calculate descriptive statistics and perform a variation coefficient analysis (variation coefficient = standard deviation/mean value × 100%). Differences in the stoichiometric traits of leaves, absorptive roots, and rhizosphere soils from *H. rhamnoides* at different stumping heights were statistically analyzed using a one-way analysis of variance (ANOVA). Significance tests with Fisher’s least significant difference (LSD) were conducted at a significance level of *p* < 0.05. To reduce errors and improve the normality of the data, the logarithm to base 10 of the N and P contents was taken. The allometric growth equation *y* = *βx^a^* was used to fit the relationships of the C, N, and P contents between the leaves and absorptive roots of *H. rhamnoides*. The logarithm on both sides of the equation was taken to obtain lg *y* = *a* lg *x* + lg *β*, where *y* and *x* represent the dependent variable and independent variable, respectively; *β* is the allometric constant (the intercept of the linear curve); and *α* is the allometric growth index (the slope of the linear curve). *α* = 1 means that *y* and *x* are in an isokinetic growth relationship, while *α* > 1 or *α* < 1 indicates an allometric growth relationship. The allometric index and constant were calculated using standardized major axis regression (SMA) based on the package smatr in R4.4.3. Nutrient stoichiometric trait maps, allometric growth maps, and related heat maps were plotted using RStudio 2024 and Origin 2021 Pro.

## 3. Results

### 3.1. Contents and Stoichiometric Changes of C, N, and P in Leaves and Absorptive Roots of H. rhamnoides

The contents and stoichiometric ratios of C, N, and P in the leaves and absorptive roots of *H. rhamnoides* after different treatments were tested via one-way ANOVA (Table 1). The C contents (LC, RC) in the leaves and absorptive roots did not differ significantly among the different treatments (*p* > 0.05). The N contents (LN, RN), P contents (LP, RP), C:N ratios (LC:LN, RC:RN), N:P ratios (LN:LP, RN:RP), and C:P ratios (LC:LP, RC:RP) in both the leaves and absorptive roots all differed significantly between different treatments (*p* < 0.05).

**Figure 2:**
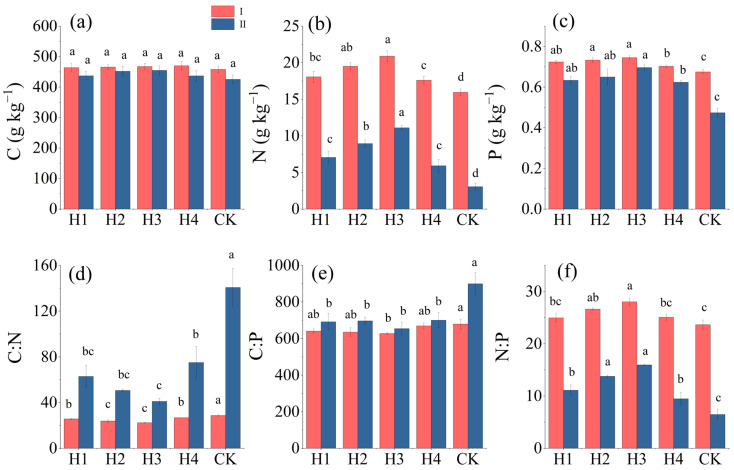
Contents (**a**–**c**) and stoichiometric ratios (**d**–**f**) of C, N, and P in leaves and absorptive roots of *H. rhamnoides* after different treatments. I: leaves. II: absorptive roots. Different lowercase letters indicate significant differences between treatments (*p* < 0.05).

The change trends in the C, N, and P contents in the absorptive roots of different treatments were basically consistent with those in the leaves (Figure 2). The ranges of LC and RC among the different treatments were 458.01–470.09 and 425.48–454.74 g·kg^−1^, respectively. LC was larger than RC after any treatment (Figure 2a). LN, RN, LP, and RP after any treatment (H1, H2, H3, or H4) were all significantly larger than those for CK (*p* < 0.05) The LN, RN, LP, and RP values among the different treatments all ranked as follows: H3 > H2 > H1 > H4 > CK (Figure 2b,c).

The LC:LN, RC:RN, LC:LP, RC:RP, LN:LP, and RN:RP values of H3 all significantly differed from those of CK (*p* < 0.05). The change trends in the ecological stoichiometric properties were basically consistent between the absorptive roots and the leaves. LC:LN, RC:RN, LC:LP, and RC:RP all first decreased and then increased after any treatment, while LN:LP and RN:RP first increased and then decreased (Figure 2). LC:LN and RC:RN of H1, H2, H3, and H4 were both significantly different from those of CK (*p* < 0.05) (Figure 2d). The LC:LP value of H3 and the RC:RP values of H1, H2, H3, and H4 were significantly lower than those of CK (all *p* < 0.05) (Figure 2e). LN:LP was greater than RN:RP in all of H1, H2, H3, and H4. The LN:LP value was greater than 16 and the RN:RP value was less than 16 after the different treatments (Figure 2f).

### 3.2. Contents of and Stoichiometric Changes in C, N, and P in Rhizosphere Soils of H. rhamnoides

The C, N, and P contents and their stoichiometric ratios in rhizosphere soils after different treatments were tested via one-way ANOVA (Table 2). The C, N, and P contents and stoichiometric characteristics of the rhizosphere soils differed significantly across the treatments (*p* < 0.05). The C and N contents and C:N ratio in rhizosphere soils all differed significantly among the treatments (*p* < 0.05), while the P content, C:P ratio, and N:P ratio in the rhizosphere soils differed very significantly among the treatments (*p* < 0.01).

**Figure 3:**
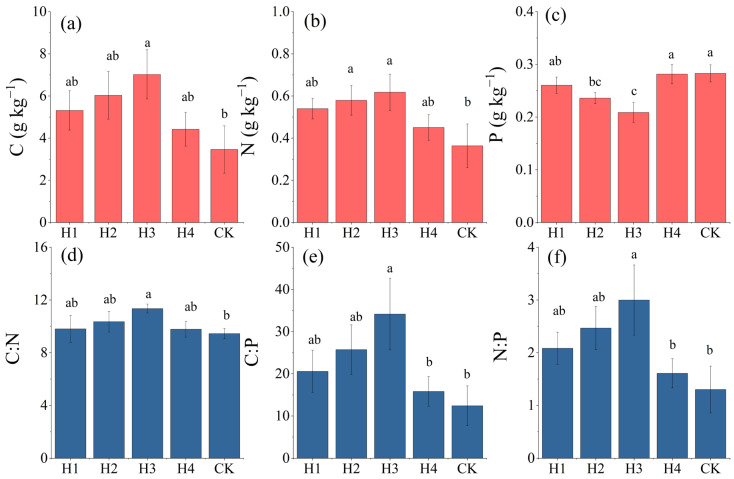
Contents (**a**–**c**) and stoichiometric ratios (**d**–**f**) of C, N, and P in rhizosphere soils of *H. rhamnoides* after different treatments. Different lowercase letters indicate significant differences between treatments (*p* < 0.05).

The contents and ecostoichiometry of C, N, and P in H3 were all significantly different from those in CK (*p* < 0.05) (Figure 3). The C contents among the different treatments varied within 3.46–7.02 g·kg ^−1^ and ranked as follows: H3 > H2 > H1 > H4 > CK. The C content of H3 increased by 1.03 times compared to that of CK (Figure 3a). The N contents of H2 and H3 were both significantly higher than that of CK (*p* < 0.05). The N content varied within 0.36–0.62 g·kg ^−1^ among the different treatments and ranked as follows: H3 > H2 > H1 > H4 > CK. The N content of H3 increased by 0.70 times compared to that of CK (Figure 3b). The P contents of H2 and H3 were both significantly higher than that of CK (*p* < 0.05). The P content varied within 0.21–0.28 g·kg ^−1^ and ranked as follows: H3 < H2 < H1 < H4 < CK (Figure 3c). The C:N, C:P, and N:P ratios of the root soils all first increased and then declined with an increment in the stumping height. The C:N, C:P, and N:P values of the various stumping treatments showed no significant differences from those of CK (*p* > 0.05), except those of H3, which all significantly differed from those of CK (*p* < 0.05). C:N, C:P, and N:P varied within 9.37–11.35 (Figure 3d), 12.42–34.17 (Figure 3e), and 1.29–2.30 (Figure 3f), respectively, across the different treatments.

### 3.3. Allometric Growth Relationship of C, N, and P Between Leaves and Absorptive Roots of H. rhamnoides

**Figure 4:**
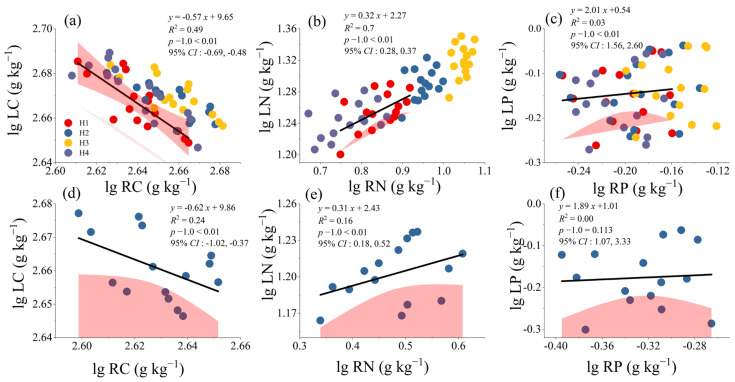
C (**a**,**d**), N (**b**,**e**), and P (**c**,**f**) allocation relationships between leaves and absorptive roots after different treatments. *p* − 1.0 represents the significance test of the slope and 1.0 at *p* = 0.05.

LP and RP of CK were found to have an isokinetic growth relationship (*p* − 1.0 > 0.05), and the C, N, and P distributions after different treatments all showed an extremely significant allometric growth relationship between the leaves and absorptive roots (*p* − 1.0 < 0.01) (Figure 4). LC and RC were found to have a very significant allometric growth relationship after any treatment (−0.57; 95% CI: −0.69, −0.48) and in CK (−0.62; 95% CI: −1.02, −0.37) (*p* < 0.01). Moreover, the allometric growth index data were all negative (Figure 4a,d), indicating a trade-off in the C distribution between the leaves and absorptive roots of *H. rhamnoides*, irrespective of stumping.

LN and RN (0.32; 95% CI: 0.28, 0.37), LP and RP (2.01; 95% CI: 1.56, 2.60) after different stumping treatments, and LN and RN in CK (0.31; 95% CI: −1.08, 0.52) all presented very significant allometric growth relationships (*p* − 1.0 < 0.01). Moreover, the allometric growth index data were all positive (Figure 4b,c,e), indicating synergy in the N and P distributions between the leaves and absorptive roots of *H. rhamnoides*, irrespective of stumping.

The allometric growth index values between LC and RC and between LN and RN were both less than 1 after any treatment (Figure 4), suggesting that the accumulation rates of LC and LN were slower than those of RC and RN. The allometric growth index value between LP and RP was larger than 1 after any treatment (Figure 4c,f), suggesting that the accumulation rate of LP is faster than that of RP.

A pairwise analysis of allometric relationships of C, N, and P between the leaves and absorptive roots of *H. rhamnoides* after different treatments was conducted (Table 3). Except for the isokinetic relationships between RN and LP for H1, H4, and CK (*p* − 1.0 > 0.05), an allometric relationship was found in all comparisons (*p* − 1.0 < 0.05). The allometric index values for LN–RC and LP–RC were negative after different treatments, indicating LN–RC and LP–RC trade-offs. Specifically, for H1, the absolute value of the allometric index was maximized for LN and RC (−1.59; 95% CI: −2.59, −0.98), and for CK, it was maximized for LP and RC (−4.63; 95% CI: −8.17, −2.62). The allometric index for LP and RN was positive after different treatments, suggesting synergy between LP and RN. Moreover, the allometric index of H3 was the largest (3.70; 95% CI: 2.15, 6.37). The absolute values of the allometric index for LN–RC and LP–RC after different treatments and for LP–RN after H2 and H3 were all greater than 1. This result indicates that the accumulation rates of LN and LP were faster than that of RC after different treatments, and the accumulation rate of LP was faster than that of RN in both H2 and H3.

### 3.4. Correlation Analysis of Allometric Data for Leaves, Absorptive Roots, and Rhizosphere Soils of H. rhamnoides

**Figure 5:**
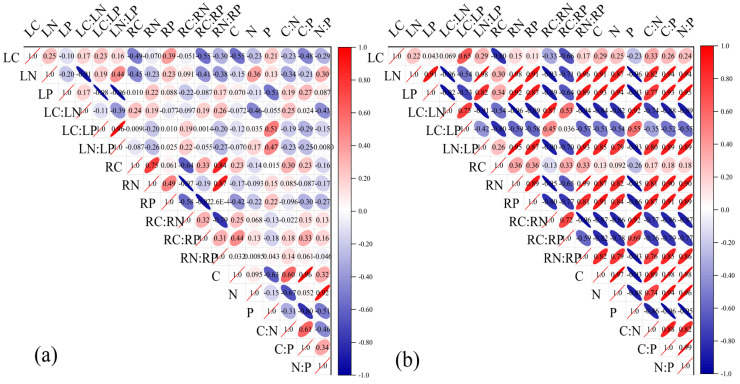
The results of a correlation analysis between C, N, and P contents and stoichiometric ratios in the leaves, absorptive roots, rhizosphere soils of non-stumped (**b**) and stumped *H. rhamnoides* (**a**).

The C, N, and P contents and correlations between stoichiometric ratios in the leaves, absorptive roots, and rhizosphere soils of *H. rhamnoides* showed significant correlations between LN and LC:LN, between LP and LC:LP or LN:LP, between LC:LP and LN:LP, between RN:RP and RC or RN, between RC:RP and RP, between C and C:P, between N and N:P, and between P and C:P (*p* < 0.05). The other correlations between C, N, and P contents and stoichiometric ratios in the leaves, absorptive roots, and rhizosphere soils were poor (Figure 5a). The correlations between C, N, and P contents and stoichiometric ratios in the leaves, absorptive roots, and rhizosphere soils of *H. rhamnoides* were generally enhanced after stumping (Figure 5b), and the positive or negative correlations of some ecological stoichiometric traits were altered. No significant correlation was found for LC or RC with any other trait (*p* > 0.05), but significant correlations were observed for all the remaining C, N, and P contents and stoichiometric ratios in the leaves, absorptive roots, and rhizosphere soils. For example, significant positive correlations were observed between LN (or RN) and LN:LP, RN:RP, C:N, C:P, and N:P (*p* < 0.05), and significant negative correlations were observed between LN (or RN) and LC:LN, LC:LP, RC:RN, and RC:RP (all *p* < 0.05).

## 4. Discussion

### 4.1. C, N, and P Contents and Stoichiometric Changes in Leaves, Absorptive Roots, and Rhizosphere Soils of H. rhamnoides

The changes in the C, N, and P contents and stoichiometric traits of the absorptive roots after different stumping treatments were basically consistent with those of the leaves (Figure 1), which reflects the collaborative response mechanism between plant organs. Absorptive roots and leaves are core plant organs for nutrient acquisition and utilization in plants; thus, their C, N, and P contents and stoichiometric traits change simultaneously as part of plants’ resource adaptation strategies [24,25].

The C contents of the leaves and absorptive roots were not significantly different among the treatments (*p* > 0.05) (Table 1), but the C contents after stumping were generally higher when compared with the non-stumped trees (Figure 2a). This outcome is closely related to the structural function of C in plants. As a major component of cell walls, the content of C is mainly controlled by genetic characteristics [26]. The small increase in C contents after stumping may be ascribed to the rapid synthesis of new tissue and the carbon sequestration caused by increased root secretion [27].

The stumping treatments significantly increased the N and P contents in the leaves and absorptive roots (*p* < 0.05) (Figure 2b,c), which is directly related to enhanced metabolic activities during plant reproduction. N is an important component of proteins and plays an essential role in plant production and photosynthesis [28,29]. P is involved in energy metabolism and nucleic acid synthesis in plants [30]. Stumping meets the rapid growth needs of the aboveground parts by breaking apical dominance, stimulating lateral bud germination, and promoting the root system to preferentially absorb N and P. Similar research results have been reported for another arid plant, *Pinus tableulaeformis* [31].

The C contents of the rhizosphere soils after stumping were significantly higher than those of CK (*p* < 0.05) (Figure 3a). This is because the activity of root systems is an important factor affecting organic carbon in rhizosphere soils [32]. Root turnover is accelerated after stumping, which promotes fine root renewal. Hence, the root system secretes more organic acids and carbohydrates [27], while the dead root system also becomes a source of soil organic carbon through decomposition [33], thereby increasing the C content. The N contents of the rhizosphere soils after stumping were significantly higher than those of CK (*p* < 0.05) (Figure 3b). A possible reason for this is that changes in the rhizosphere soil N content are affected by the nitrogen fixation ability of root nodules in *H. rhamnoides* [34]. The activities of symbiotic nitrogen-fixing bacteria with *H. rhamnoides* were enhanced after stumping, thereby increasing the soil N supply. In contrast, the P content of the rhizosphere soils was significantly reduced after stumping (*p* < 0.05) (Figure 3c). This may be because soil P is dominated by insoluble phosphates, and P is easily fixed in the soils in feldspathic sandstone areas owing to their high clay content [35]. In addition, the active absorption of P by stumped plants leads to the depletion of available P [36], so the P content of the rhizosphere soil after stumping was significantly reduced due to absorption by the root system.

C:N and C:P reflect the carbon assimilation ability of plants during the absorption of nutrient elements and indicate the use efficiency of these elements. Generally, low C:N and C:P values suggest a fast-growing plant [37]. The C:N and C:P values for the leaves and absorptive roots of stumped *H. rhamnoides* were lower than those for CK, and the LC:LN, RC:RN, LC:LP, and RC:RP values of H3 were significantly different from those of CK (*p* < 0.05) (Figure 2). These results indicate that stumping accelerated biomass accumulation by promoting N and P absorption and reducing the nutrient cost per unit of carbon consumption, reflecting an improvement in the resource utilization efficiency of the plants. *H. rhamnoides* with a stumping height of 15 cm enjoyed a faster growth rate.

Reportedly, the N:P ratio indicates N or P restriction in plants. N:P < 14 and N:P > 16 usually suggest N restriction and P restriction, respectively, and a value between 14 and 16 indicates the restriction of both N and P [38,39]. In our study, LN:LP was greater than 16 after any treatment, indicating that the leaves were mainly restricted by P. This indication is consistent with the law that plants in arid areas are generally restricted by P. The RN:RP ratios after treatments H1, H2, H3, H4, and CK were 11.11, 13.77, 15.96, 9.47, and 6.46, respectively (Figure 2f). Specifically, the RN:RP value of H3 was within 14–16, indicating that the absorptive roots of *H. rhamnoides* at a stumping height of 15 cm were subject to the co-restriction of N and P. RN:RP was less than 14 after any of the other treatments. These results suggest that the absorptive roots were mainly limited by N. A possible reason why the absorptive roots after different stumping treatments were limited by N is that the activities of nitrogen-fixing bacteria lagged behind the plant growth demand after stumping, making N a short-term limiting factor. *H. rhamnoides* was already in a recession period, and the nitrogen-fixing ability of its root system failed to meet its reproductive needs.

Soil C:N:P is the ratio of the total masses of C, N, and P in soil organic matter or other components; C:N reflects the rate of soil biological activity, and C:P and N:P reflect restricted states of soil nutrients [40]. The C:N, C:P, and N:P ratios of the rhizosphere soils first rose and then dropped with an increment in the stumping height. The C:N, C:P, and N:P ratios in the rhizosphere soils after stumping were all larger than those for CK (Figure 3). The rise in the rhizosphere soil C:N ratio implies that the organic carbon content of the root soils was larger than their N content after stumping, so the carbon supply was sufficient. The rise in C:P and N:P in the rhizosphere soils indicates a drop in P availability, resulting in relative enrichment of C and N.

### 4.2. Correlation Between Stoichiometric Ratios and Allometric Growth in Leaves, Absorptive Roots, and Rhizosphere Soils of H. rhamnoides

The functional and metabolic activities of plant organs are important factors that restrict nutrient allocation among the organs [41]. The differences in allometric index values among different organs indicate the nutrient allocation patterns and functional balance [42]. In our study, LC and RC were found to be in a very significant allometric growth relationship after any treatment (−0.57; 95% CI: −0.69, −0.48) and in CK (−0.62; 95% CI: −1.02, −0.37) (*p* − 1.0 < 0.01). Moreover, the C contents in the leaves and absorptive roots of *H. rhamnoides* presented a trade-off relationship (allometric index < 0), indicating competition in C allocation between the two organs. This competition occurs because, under drought stress in the feldspathic sandstone area, plants enter a state of reduced carbon consumption in their roots, reducing water loss, and allocate more resources to the leaves for photosynthesis and stress resistance. At the same time, the carbon fixation capacity of the leaves is guaranteed to maintain survival under drought stress. This trade-off is the evolutionary result of the plants’ long-term adaptation to environmental stress [43]. The allometric index for LC and RC changed from −0.62 to −0.57 after stumping (Figure 4a,d), which may be attributed to the reproduction strategy of *H. rhamnoides* after stumping. Specifically, the plants quickly restored their nutrient-absorbing capacity by preferentially supporting root regeneration and thereby achieved a balance between growth and stress adaptation.

LN and RN (0.32; 95% CI: 0.28, 0.37), LP and RP (2.01; 95% CI: 1.56, 2.60) after different stumping treatments, and LN and RN in CK (0.31; 95% CI: −1.08, 0.52) each showed a very significant allometric relationship (*p* − 1.0 < 0.01). Moreover, N and P in the leaves and absorptive roots of *H. rhamnoides* exhibited synergy (allometric index > 0, Figure 4), reflecting functional complementarity. A synergistic increase in N contents in the leaves and absorptive roots indicates that plants can maximize their resource utilization efficiency by synchronously improving the synthesis of photosynthetic enzymes and protein absorption in their roots (e.g., nitrate reductase) [44]. Similarly, the synergistic allocation of P is closely related to nucleic acid synthesis [45] and reflects the energy demand of plants during reproduction. LN and RN (0.31) in non-stumped *H. rhamnoides* (Figure 4e) were synergistically allocated to balance photosynthetic and absorption capacities, so as to adapt to the long-term nutrient restriction environment. The synergistic index increased after stumping (0.32) (Figure 3b), indicating that the plants’ reproduction efficiency was improved by the enhanced nitrogen fixation ability of their root nodules and their improved N utilization efficiency and rhizosphere environment. This change is the adaptive response of *H. rhamnoides* to stumping and manifests as rapid recovery. The LP and RP of CK grew isokinetically (1.89) (*p* − 1.0 > 0.05) (Figure 4f), indicating that the P distribution between the leaves and absorptive roots reached a steady state. The increase in the allometric index after stumping (2.01). (Figure 4c) indicates the reproduction demand that forced the plants to break this steady state and preferentially allocate P to the leaves to accelerate photosynthesis.

The accumulation rates of C and N in the leaves were slower than those in the absorptive roots (allometric index < 1) (Figure 4), which is due to an adaptive strategy achieved through organ functional differentiation and optimized resource allocation. The absorptive roots preferentially acquire N during the reproduction period and accelerate carbon metabolism, reflecting the efficient use of finite resources by plants [46]. The P accumulation rate in the leaves was faster than that in the roots (allometric index > 1). The reason for this may be that photosynthesis, energy metabolism activities, and nucleic acid synthesis in plants require a continuous P supply [31], so that the leaves’ P demand is more dependent on root absorption.

Stumping significantly changed the allometric index by breaking the original resource balance. Specifically, the allometric index between LP and RN in H3 was as high as 3.70 (Table 3), indicating that synergistic allocation of P between the leaves and roots was strengthened. Consequently, the P content was the lowest in rhizosphere soils (Figure 3c). A possible reason for this is that stumping enabled improved P absorption efficiency in the absorptive roots so as to support the rapid reconstruction of the leaf photosynthetic mechanism, thus forming a positive feedback cycle of “P absorption/photosynthetic utilization” [47] and achieving efficient circulation of P. The above change may be the key mechanism for the optimal growth observed after the H3 treatment. The value of the allometric index reflects the adaptation strategy of plants to the environment. Our results showed that the allometric index between LN and RC was negative after any treatment (Table 3), indicating a trade-off between N allocation and root carbon investment. In the feldspathic sandstone area, this trade-off may achieve a balance between survival and growth under limited resources by reducing root carbon consumption to save energy and distributing more N to the leaves to maintain photosynthetic efficiency [48].

The correlations between the C, N, and P contents and stoichiometric ratios in the leaves, absorptive roots, and rhizosphere soils of non-stumped *H. rhamnoides* were generally low (Figure 5a) but were basically enhanced after stumping (Figure 5b). This change implies that plants adjust their strategy in response to interference. The correlations of nutrients between the organs of non-stumped *H. rhamnoides* and rhizosphere soils were low, owing to long-term resource limitations. After stumping, the nutrient correlations among the leaves, roots, and rhizosphere soils were generally enhanced as the plants redistributed resources through compensatory growth. This is because the original nutrient balance was broken after stumping, forcing plants to establish a new allocation pattern, and root secretions changed the rhizosphere microenvironment, promoting nutrient circulation. The C contents of the leaves and absorptive roots were not significantly correlated with other indicators (*p* > 0.05). The lack of correlations between LC or RC and other traits is attributed to the structural stability and independent regulation of carbon [26]. The enhanced correlations between N, P, and their stoichiometric ratios resulted from the joint effect of strengthened metabolic activities, rhizosphere feedback, and allometric growth.

In sum, stumping can improve the resource use efficiency of *H. rhamnoides* and change the nutrient dynamic effect in rhizosphere soils. In ecosystems with similar stress conditions, as in feldspathic sandstone areas, managers can adopt a stumping strategy to optimize nutrient allocation in target species, thereby improving their growth and adaptability. Our results provide a theoretical basis for shrub or perennial plant management in arid or resource-constrained environments.

## 5. Conclusions

The effects of stumping heights on the stoichiometric traits and allometric growth in the leaves, absorptive roots, and rhizosphere soils of *H. rhamnoides* growing in feldspathic sandstone areas were explored. The stumped *H. rhamnoides* had a faster growth rate. Except for LC and RC, all of the nutrient stoichiometric traits were significantly impacted by the stumping treatments, and the optimal stumping height was 15 cm. Moreover, the changes in the contents and ecological stoichiometric ratios of C, N, and P in the absorptive roots after different treatments were basically consistent with those in the leaves. A trade-off relationship between LC and RC and synergistic relationships between LN and RN and between LP and RP were found in *H. rhamnoides* after different treatments. The accumulation rates of LC and LN were slower than those of RC and RN. The correlations between C, N, and P contents and stoichiometric ratios in the leaves, absorptive roots, and rhizosphere soils of *H. rhamnoides* were generally enhanced after stumping, and the positive or negative correlations of some ecological stoichiometric features were altered in the unstumped parts. Thus, during vegetation recovery, *H. rhamnoides* forests in feldspathic sandstone areas should be stumped to a height of 15 cm to improve their decaying status.

## Figures and Tables

**Table 1:** C, N, and P contents in leaves and absorptive roots of *H. rhamnoides*, and sources of stoichiometric changes.

Traits	*df* (Degrees of Freedom)	*SS* (Sum of Squares)	*MS* (Mean Square)	*F*	*p*
LC (leaf C content, g kg^−1^)	4	250.77	62.69	0.46	0.766
LN (leaf N content, g kg^−1^)	4	42.43	10.61	28.79	0.0001
LP (leaf P content, g kg^−1^)	4	0.0092	0.0023	24.77	0.0001
LC:LN (leaf LC:LN ratio)	4	72.14	18.035	47.78	0.0001
LC:LP (leaf LC:LP ratio)	4	5991.52	1497.885	4.38	0.0264
LN:LP (leaf LN:LP ratio)	4	34.41	8.60	18.39	0.0001
RC (absorptive root C content, g kg^−1^)	4	1765.67	441.42	1.77	0.212
RN (absorptive root N content, g kg^−1^)	4	111.38	27.84	69.15	0.0001
RP (absorptive root P content, g kg^−1^)	4	0.084	0.021	38.53	0.0001
RC:RN (absorptive root LC:LN ratio)	4	18,614.59	4653.65	40.93	0.0001
RC:RP (absorptive root LC:LP ratio)	4	113,709.42	28,427.36	15.43	0.0003
RN:RP (absorptive root LN:LP ratio)	4	163.66	40.91	58.095	0.0001

**Table 2:** C, N, and P contents in rhizosphere soils of *H. rhamnoides*, and sources of stoichiometric changes.

Traits	*df* (Degrees of Freedom)	*SS* (Sum of Squares)	*MS* (Mean Square)	*F*	*p*
C (rhizosphere soil C content, g kg^−1^)	4	22.91	5.73	5.30	0.0149
N (rhizosphere soil N content, g kg^−1^)	4	0.13	0.032	5.43	0.0138
P (rhizosphere soil P content, g kg^−1^)	4	0.012	0.003	11.46	0.0009
C:N (rhizosphere soil C:N ratio)	4	6.66	1.67	3.63	0.0446
C:P (rhizosphere soil C:P ratio)	4	880.0021	220.0005	6.63	0.0071
N:P (rhizosphere soil N:P ratio)	4	5.46	1.37	6.99	0.0059

**Table 3:** Allometric relationships of C, N, and P contents in leaves and absorptive roots of *H. rhamnoides* after different treatments.

Index (lg *y* − lg *x*)	Treatment	*n*	*R* ^2^	*p*	Slope	95% CI	Intercept	*p* − 1.0	Type
LN–RC	H1	15	0.30	<0.001 **	−1.59	−2.59, −0.98	12.58	<0.001 **	A
	H2	15	0.02	<0.01 **	−1.24	−2.19, −0.71	10.57	<0.001 **	A
	H3	15	0.32	<0.001 **	−1.34	−2.16, −0.83	11.26	<0.001 **	A
	H4	15	0.25	<0.01 **	−1.21	−1.99, −0.74	10.22	<0.01 **	A
	CK	15	0.19	<0.01 **	−1.48	−2.49, −0.89	11.75	<0.001 **	A
LP–RC	H1	15	0.01	<0.01 **	−4.49	−7.92, −2.55	26.98	<0.001 **	A
	H2	15	0.03	<0.01 **	−4.26	−7.45, −2.43	−26.36	<0.05 *	A
	H3	15	0.01	<0.01 **	−4.26	−7.51, −2.42	25.79	<0.001 **	A
	H4	15	0.00	<0.01 **	−3.86	−6.80, −2.19	23.09	<0.001 **	A
	CK	15	0.00	<0.01 **	−4.63	−8.17, −2.62	27.63	<0.001 **	A
LP–RN	H1	15	0.04	<0.01 **	1.35	0.77, 2.36	−2.96	0.359	I
	H2	15	0.08	<0.01 **	2.41	1.39, 4.17	5.60	<0.05 *	A
	H3	15	0.09	<0.01 **	3.70	2.15, 6.37	−9.21	<0.05 *	A
	H4	15	0.03	<0.01 **	1.20	0.68, 2.09	−2.48	0.551	I
	CK	15	0.04	<0.01 **	0.96	0.55, 1.67	−1.46	0.88	I

*p* − 1.0 represents the significance test of the slope and 1.0 at *p* = 0.05; * *p* < 0.05; ** *p* < 0.01; A indicates an allometric relationship; I indicates an isokinetic relationship.

## Data Availability

All datasets generated and/or analyzed during the current study are included in this article.
